# *USP6*-associated soft tissue tumors with bone metaplasia: Clinicopathologic and genetic analysis and the identification of novel *USP6* fusion partners

**DOI:** 10.3389/fonc.2022.1065071

**Published:** 2023-01-16

**Authors:** Yahan Zhang, Yan Qiu, Xianliang Zhang, Xin He, Chen Chen, Min Chen, Hongying Zhang

**Affiliations:** Department of Pathology, West China Hospital, Sichuan University, Chengdu, China

**Keywords:** *USP6* rearrangement, myositis ossificans, fibro-osseous pseudotumor of digits, soft tissue aneurysmal bone cyst, fasciitis ossificans, differential diagnosis, *USP6*-associated neoplasms

## Abstract

**Introduction:**

Among those tumors with consistent USP6 rearrangement, some arise from soft tissue and show bone metaplasia, including myositis ossificans (MO), fibro-osseous pseudotumor of digits (FOPD), soft tissue aneurysmal bone cyst (ST-ABC) and fasciitis ossificans (FO). These lesions are easily confused with malignancies because they show a rapid growth rate and brisk mitoses. Here, we aim to clarify the clinicopathologic and genetic characteristics of this entity and analyze the correlations among the different subtypes in one of the largest cohorts.

**Materials and Methods:**

The clinicopathologic features of 73 cases of MO, FOPD, ST-ABC and FO diagnosed at West China Hospital, Sichuan University from January 2010 to December 2021 were retrospectively analyzed. Forty-three undecalcified samples were analyzed by systematic genetic studies, including fluorescence in situ hybridization (FISH), reverse transcription polymerase chain reaction (RT-PCR), Sanger sequencing and next-generation-based sequencing were performed.

**Results:**

This series included 40 males and 33 females aged 2 to 80 years old (median: 31 years). FOPD occurred in extremal soft tissue, while lower extremities (38/58, 65.5%) were the most commonly involved lesions in the other three subgroups. Histologically, proliferative myofibroblasts/fibroblasts with varying degrees of osteoid tissue were present. Fluorescence in situ hybridization (FISH) results indicated that 22 cases (22/27, 81.5%) were positive for USP6 rearrangement, and 5 cases were negative. Among those cases with positive FISH results, 18 underwent reverse transcription-polymerase chain reaction (RT-PCR) detection that successfully detected common USP6 fusion types. Thirteen cases showed COL1A1::USP6 fusion, one showed MYH9::USP6 fusion, and 4 were negative for common fusion types. Next-generation-based sequencing technology was performed on two lesions with negative RT–PCR results and novel fusion partners SNHG3 and UBE2G1 were discovered.

**Conclusions:**

Our findings revealed that COL1A1 is the most common fusion partner in this entity, unlike primary aneurysmal bone cysts and nodular fasciitis. Notably, we believed that FO may demonstrate more similar clinicopathologic and genetic manifestations with MO/FOPD and ST-ABC instead of nodular fasciitis for involving lower limbs most frequently and showing recurrent COL1A1::USP6 fusion. Additionally, this study also found two novel USP6 fusion partners, which further expanded our knowledge of this neoplastic spectrum.

## Introduction

The ubiquitin-specific protease 6 gene (*USP6*), also known as Tre-2, encodes chromosome 17p13.2 (1). In 2004, Oliveira and colleagues found recurrent *USP6* rearrangement in primary aneurysmal bone cysts (ABCs) (63%) (2). Subsequent studies confirmed that *CDH11* was the most common fusion partner for *USP6* in primary ABC (2, 3). In 2011, Erickson-Johnson et al. recognized recurrent *USP6* rearrangement and the most common fusion partner *MYH9* in nodular fasciitis (NF) (4). Thereafter, these two entities were included as *USP6*-associated neoplasms (1). In recent years, a growing number of molecular genetic studies (including research from our group) have expanded the families of *USP6*-associated neoplasms. The family also includes (1) variants of NF: cranial fasciitis (CF), intravascular fasciitis (IVF) and fasciitis ossificans (FO); and (2) other spindle cell neoplastic lesions: fibroma of tendon sheath (FTS), benign infiltrative myofibroblastic neoplasms, myositis ossificans (MO), fibro-osseous pseudotumor of digits (FOPD) and soft tissue aneurysmal bone cyst (ST-ABC) (5–14).

Notably, *USP6*-associated neoplasms are characterized by proliferative myofibroblasts/fibroblasts with or without metaplasia of osteoid components. In this family, many tumors are classic pseudosarcomatoid lesions, which are easily confused with malignancies in the diagnostic process, especially for those with bone metaplasia. Our group has previously conducted some related studies on NF and its variants (some results have been published) (11, 15). This study will focus on *USP6*-associated soft tissue tumors with bone metaplasia.

*USP6*-associated soft tissue tumors with bone metaplasia predominantly consist of MO, FOPD, ST-ABC and FO. MO and FOPD have been previously recognized as tumors belonging to the same spectrum from the perspective of morphology, and recent studies have further confirmed the conclusion from the perspective of genetics for identifying consistent *USP6* rearrangements in both MO and FOPDs (8, 9). Recent research has revealed that the most common fusion partner in MO and FOPD is *COL1A1 (* 10), which is different from the primary ABC and NF. Recently, clinicopathological features and genetic changes similar to MO/FOPD have been identified in ST-ABC, while the 2020 WHO classification of soft tissue and bone tumors and some recent researchers have suggested that rare ST-ABC, MO and FOPD should be classified into the same subclass of *USP6*-associated tumors (5, 16). Notably, FO also showed ossifying components in morphology, but there are only a few studies with a small sample size on these tumors, Further studies are needed to determine whether there is a closer affinity between FO and MO, FOPD, and ST-ABC.

Based on the aforementioned background, this study will include one of the largest cohorts of MO, FOPD, ST-ABC, and FO cases to further clarify the clinicopathologic and genetic characteristics of this entity and analyze the correlations and differences among different subtypes.

## Materials and methods

### Case selection

This study was approved by the Ethics Committee on Biomedical Research, West China Hospital of Sichuan University (No. 793, 2021). A SNOMED search of the hospital surgical pathology files from January 2010 to December 2021 identified 73 *USP6*-associated soft tissue tumors with bone metaplasia, which included 44 MO cases, 15 FOPD cases, 12 FO cases and 2 ST-ABC cases. Clinical, pathological, and follow-up information was collected from clinical records and pathology reports. Follow-up information was collected by telephone interviews. The follow-up duration was calculated from the date of the first surgery to the date of recurrence, death, or last follow-up.

### Histologic review

Hematoxylin and eosin-stained and immunohistochemically stained slides were obtained from the surgical and pathological bank of the hospital, and all cases were reviewed independently by 2 experienced pathologists (Hongying Zhang and Xianliang Zhang) with expertise in soft tissue and bone tumor pathology and 1 general surgical pathologist (Yahan Zhang).

### Fluorescence *in situ* hybridization

Fluorescence in situ hybridization (FISH) was conducted on formalin-fixed, paraffin-embedded (FFPE) tissue sections using a *USP6* break-apart probe (Lbp Medicine Science & Technology, Guangzhou, China) following the manufacturer-provided instructions for 43 cases (including 24 MOs, 6 FOPDs, 11 FOs and 2 ST-ABCs) with available undecalcified tissues. Thirty decalcified cases did not perform FISH because the strong acid decalcification method routinely used would damage the sample DNA. Tumor samples were evaluated by two pathologists in 100 neoplastic cells in a blind fashion using an Olympus BX53 fluorescence microscope (Japan). A red-green split signal pattern was considered positive for *USP6* gene rearrangement if the distance between the green and red signals was greater than the diameter of any two signals. A case was considered positive for *USP6* rearrangement when 10% or more counted cells showed red-green split signals.

### Reverse transcription-polymerase chain reaction and Sanger sequencing

Total RNA was extracted using the miRNeasy FFPE Kit (Qiagen, Hilden, Germany), and the concentration and quality of RNA were measured using NanoDrop Microvolume Spectrophotometers (Thermo Fisher Scientific, Massachusetts, USA). cDNA was synthesized using the PrimeScript RT reagent kit (Takara, Tokyo, Japan). All polymerase chain reactions (PCRs) were performed for 40 cycles using a TB Green™ Premix Ex Taq™ II kit (Takara, Tokyo, Japan) with the following cycle conditions: denaturation at 94°C for 40 s, annealing at 50~60°C for 30 s, and extension at 72°C for 30 s using primers for commonly reported *USP6* fusion genes, including *MYH9::USP6, CDH11::USP6, COL1A1::USP6, SEC31A::USP6, RUNX2::USP6, PAFAH1B1::USP6, PPP6R3::USP6* and *COL1A2::USP6* (**Supplemental Table 1**). Ten microliters of each amplified product were subjected to 2% agarose gel electrophoresis and photographed by a Bio-Rad imager for visualization. Sanger sequencing was performed to verify the positive reverse transcription-polymerase chain reaction (RT-PCR) products.

### Next-generation-based technology

The FFPE tissue was sent to the sequencing core (242 gene DNA panels, Yousu™, OrigiMed, Shanghai, China). DNA was extracted from FFPE tissues using the QIAamp DNA FFPE Tissue Kit according to the manufacturer’s protocol (Qiagen, Hilden, Germany). A total of 0.5 μg of DNA per sample was applied as input for the DNA library preparations. Assays were performed using an Illumina MiSeq Platform (Illumina, San Diego, CA, USA) following the manufacturer’s recommendations.

## Results

### Clinical findings

This subgroup included 40 males and 33 females with diagnosed ages ranging from 2 to 80 years old (median: 31 years). Most cases manifested as a painful mass or swelling lesion, and the duration of these symptoms was 0.2 to 96 months (median: 1 month) in 66 patients with available data. Injury information was available for 48 patients, among which 14 (29.2%) had a history of trauma at the lesion site. Except for FOPD occurring in the soft tissues of the extremities (12 fingers and 3 toes), the lower limbs (38/58, 65.5%) were the most commonly involved sites, followed by the upper limbs (9/58, 15.5%), trunk (9/58, 15.5%), and head and neck (2/58, 3.5%) in MO, ST-ABC and FO. The range of maximum tumor diameter in 59 cases with available information was 0.5 cm to 20 cm (median: 5.0 cm).

**Figure 1:**
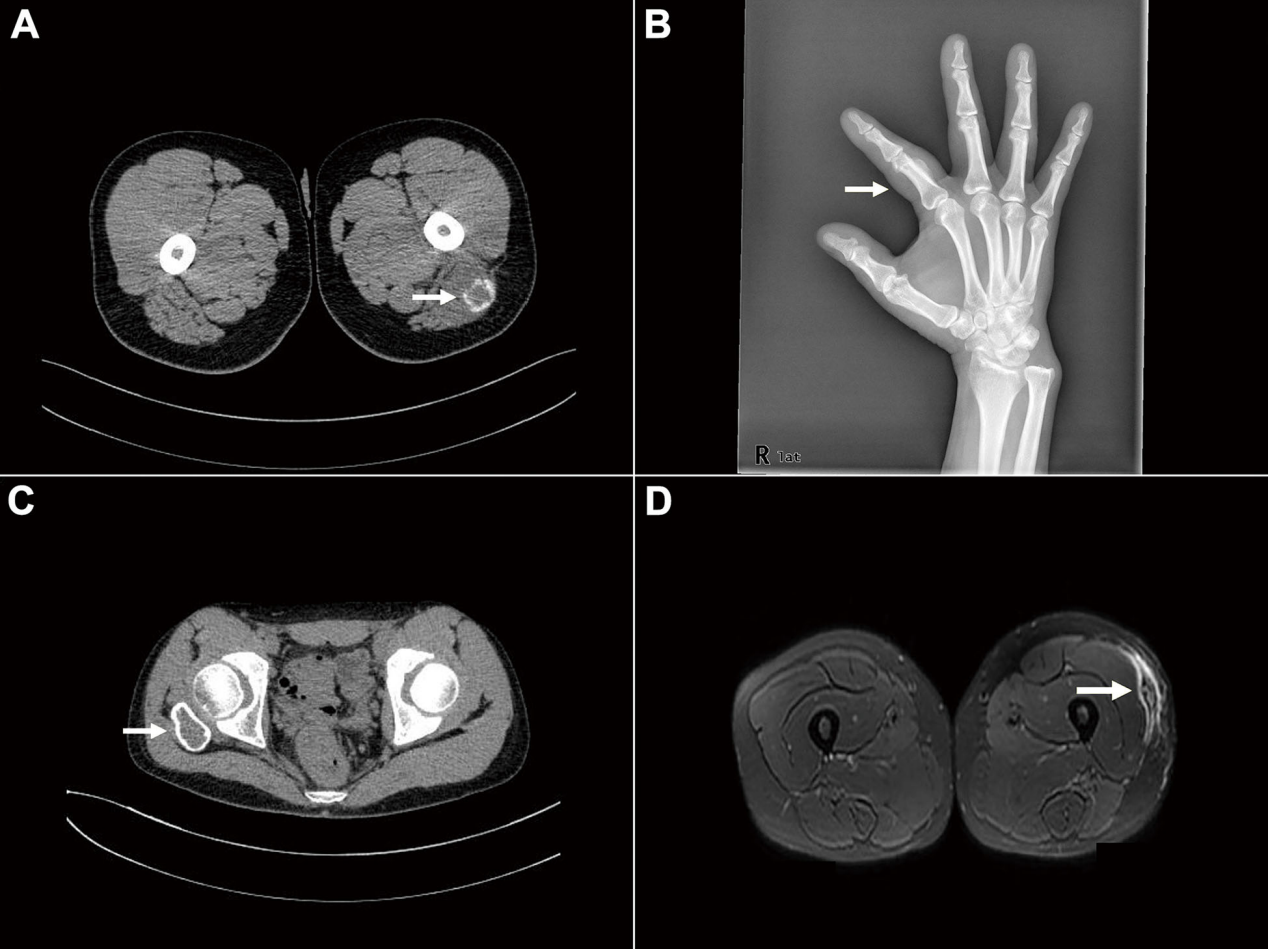
Imaging findings of *USP6*-associated soft tissue tumors with osteoid tissue. **(A)** CT showed a soft tissue density mass in the muscle, with well-defined boundaries and circumferentially circular density (arrow) (Case 33). **(B)** X-rays demonstrated a soft tissue mass in the right finger with calcification (arrow) (Case 54). **(C)** CT showed mixed-density shadows with clear boundaries around the right hip (arrow) (Case 60). **(D)** MRI showed mixed signals in the subcutaneous fat layer of the left thigh, with clear boundaries (arrow) (Case 63).

Imaging data were available for 38 cases. MO was often in the deep muscle, FOPD was predominantly located in the subcutaneous soft tissue of the fingers and toes, ST-ABC was located in the deep soft tissue, and FO was essentially confined to the subcutaneous fat layer. Tumor boundary information was obtained in 30 cases, and most of them (21/30, 70.0%) were well circumscribed on imaging. MRI is mainly manifested as a mass with an abnormal signal in the intramuscular or subcutaneous tissues. CT and/or X-ray usually showed a slightly low-density mass in the soft tissue, with mostly high-density calcification in or around the mass (15/25, 60.0%) (**Figures 1A–D**).

### Pathological findings

**Figure 2:**
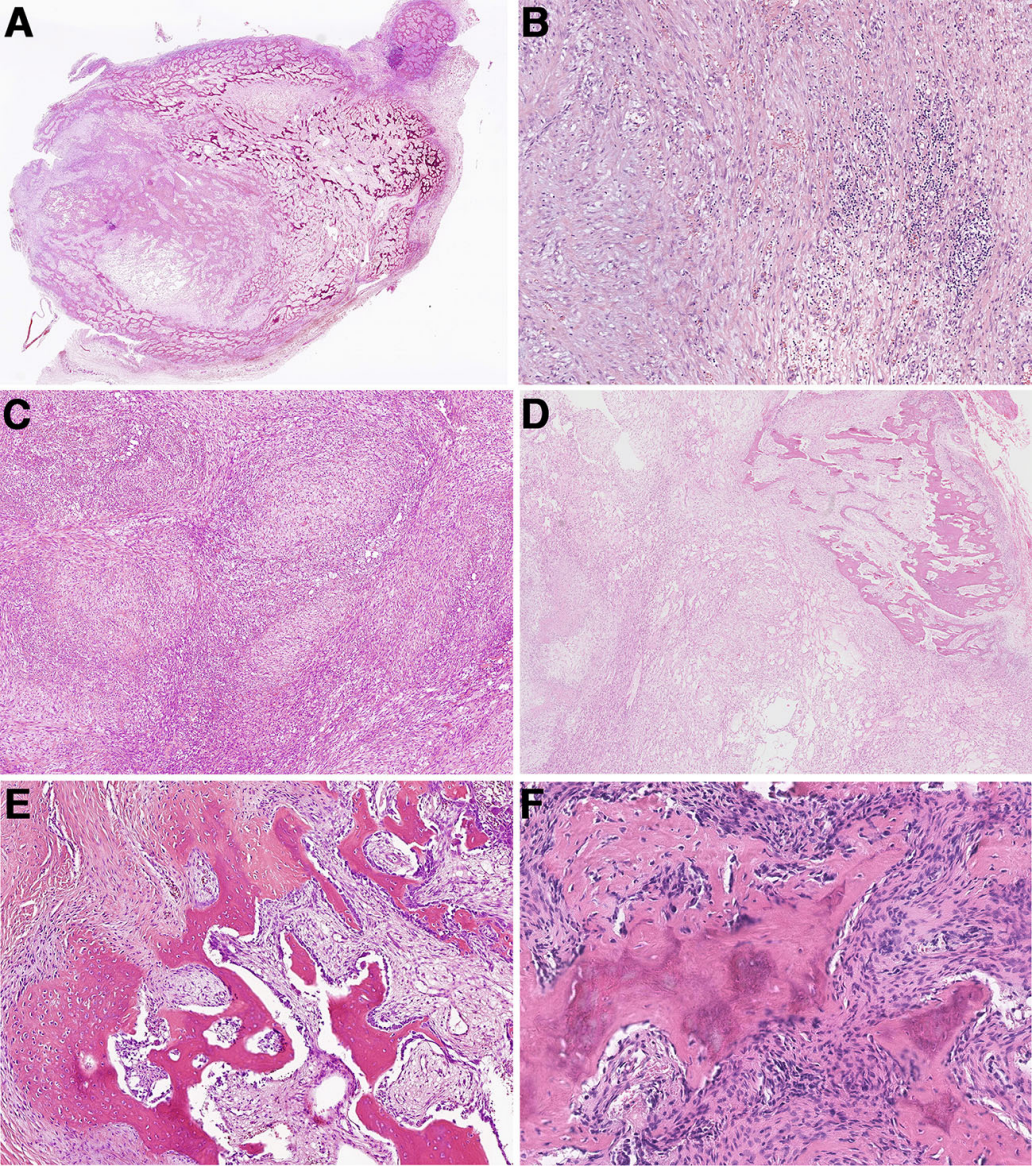
Histopathologic characteristics of myositis ossificans and fibro-osseous pseudotumors of digits. **(A)** Most myositis ossificans cases were well circumscribed with a typical zonation pattern (Case 33). **(B)** Myofibroblasts/fibroblasts were arranged in bundles in the myxoid stroma, accompanied by extravasated red blood cells and chronic inflammatory cells, showing NF-like morphology (Case 33). **(C)** Extensive NF-like morphology was observed in one myositis ossificans case (Case 42). **(D)** The tumor formed obvious zonal distribution focally (Case 42). **(E)** Osteoid components were mature and disorderly in fibro-osseous pseudotumors of digits (Case 46). **(F)** Immature osteoid components were observed scattered among the proliferative myofibroblast/fibroblast (Case 57).

**Figure 3:**
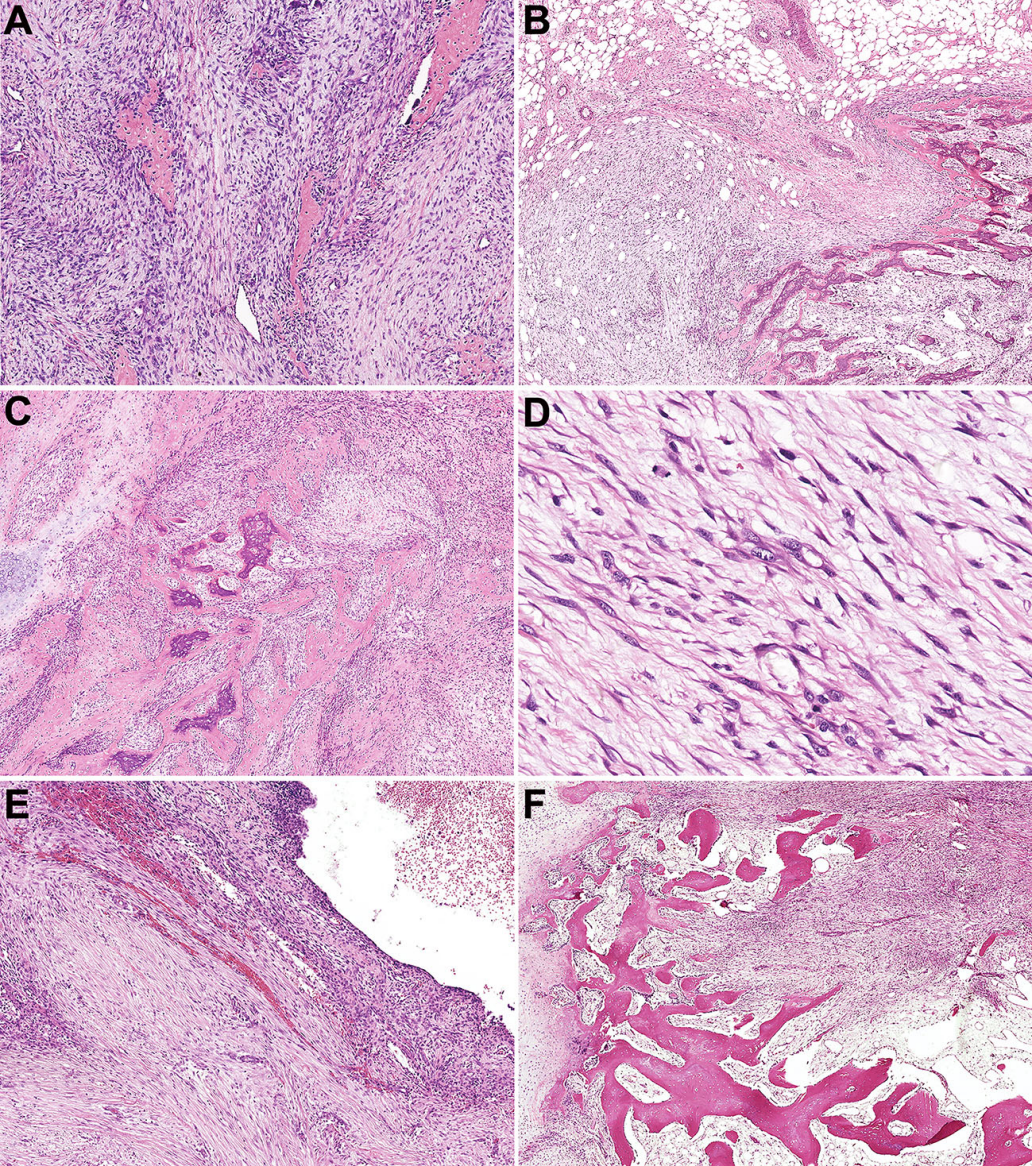
Histopathologic characteristics of fasciitis ossificans and soft tissue aneurysmal bone cyst. **(A)** Osteoid tissues were distributed disorderly in an NF-like background in fasciitis ossificans (Case 66). **(B)** In one fasciitis ossificans case, the tumor was locally invasive with a reverse zonation structure. (Case 63). **(C)** Myofibroblasts/fibroblasts cells were hyperplasia with mild atypia, and immature osteoid tissue was scattered among them. (Case 63). **(D)** Mitotic figures with no atypia were presented in myofibroblasts/fibroblasts (Case 63). **(E)** Soft tissue aneurysmal bone cyst showed a cystic cavity with hyperplastic myofibroblasts/fibroblasts and focal osteoid tissue in the cystic septum (Case 60). **(F)** In one soft tissue aneurysmal bone case, MO-like zone structure was observed and bone shell was formed around the tumor (Case 60).

Histologically, the basic lesions of this entity are myofibroblast/fibroblast proliferation with varying degrees of osteoid components. The myofibroblasts/fibroblasts may show mild atypia. Osteoblasts were often found around the mature and braided bone, and multinucleated giant cells were shown in some areas. Some (29/71, 40.1%) lesions were accompanied by cartilage metaplasia. Varying numbers of mitoses with no atypia can be observed (0~20/10 HPFs). In those MO lesions, most cases demonstrated an ill-defined intramuscular mass. Microscopically, a zonation pattern, noted as transitions from the central immature osteoid component gradually to the peripheral mature trabecular bone, can be observed in most cases (**Figure 2A**). Part of MO cases (34.1%, 15/44) showed NF-like morphology in some foci, among which myofibroblasts/fibroblasts arranged as cell culture pattern, varying degree of myxoid change, extravasated erythrocytes, infiltrating inflammatory cells (**Figure 2B**). Extremely rare cases displayed extensive and prominent NF-like morphology (**Figure 2C**) and focal osteoid components with zonation patterns (**Figure 2D**). In terms of FOPD, the morphology was similar to MO; however, osteoid components in most FOPD cases showed a haphazard pattern instead of an obvious zonation pattern (**Figures 2E, F**). Most FO had classical NF-like morphology (10/12, 83.8%), and osteoid tissue of different maturation degrees were distributed haphazardly in tumors (**Figure 3A**). Extremely rare FO cases showed local infiltration and reverse zonal distribution that is similar to osteosarcoma (**Figure 3B**). The hyperplastic myofibroblasts/fibroblasts cells showed mild atypia, and immature osteoid tissue was scattered among them (**Figure 3C**). Besides, brisk mitosis with no atypia could be observed (**Figure 3D**). ST-ABC featured blood-filled cyst formation (**Figure 3E**), and MO-like morphology with a zonation structure was also found in some regions of one ST-ABC in this group (**Figure 3F**).

Pertinent immunohistochemical markers were performed for diagnosis in some cases. Smooth muscle actin (SMA) showed diffuse expression in all detected cases (21/21, 100%), six cases exhibited MSA expression (6/8, 75.0%), and expression of SATB2 was present in the bone metaplasia area (8/8, 100%). Lacked expression of desmin in 19 cases (19/20, 95.0%). S-100 protein (16/16, 100%), β-catenin (8/8, 100%) and MDM2 (8/8,100%) expression were consistently absent. The MIB-I positive index ranged from 1 to 30%.

### Genetic findings

**Figure 4:**
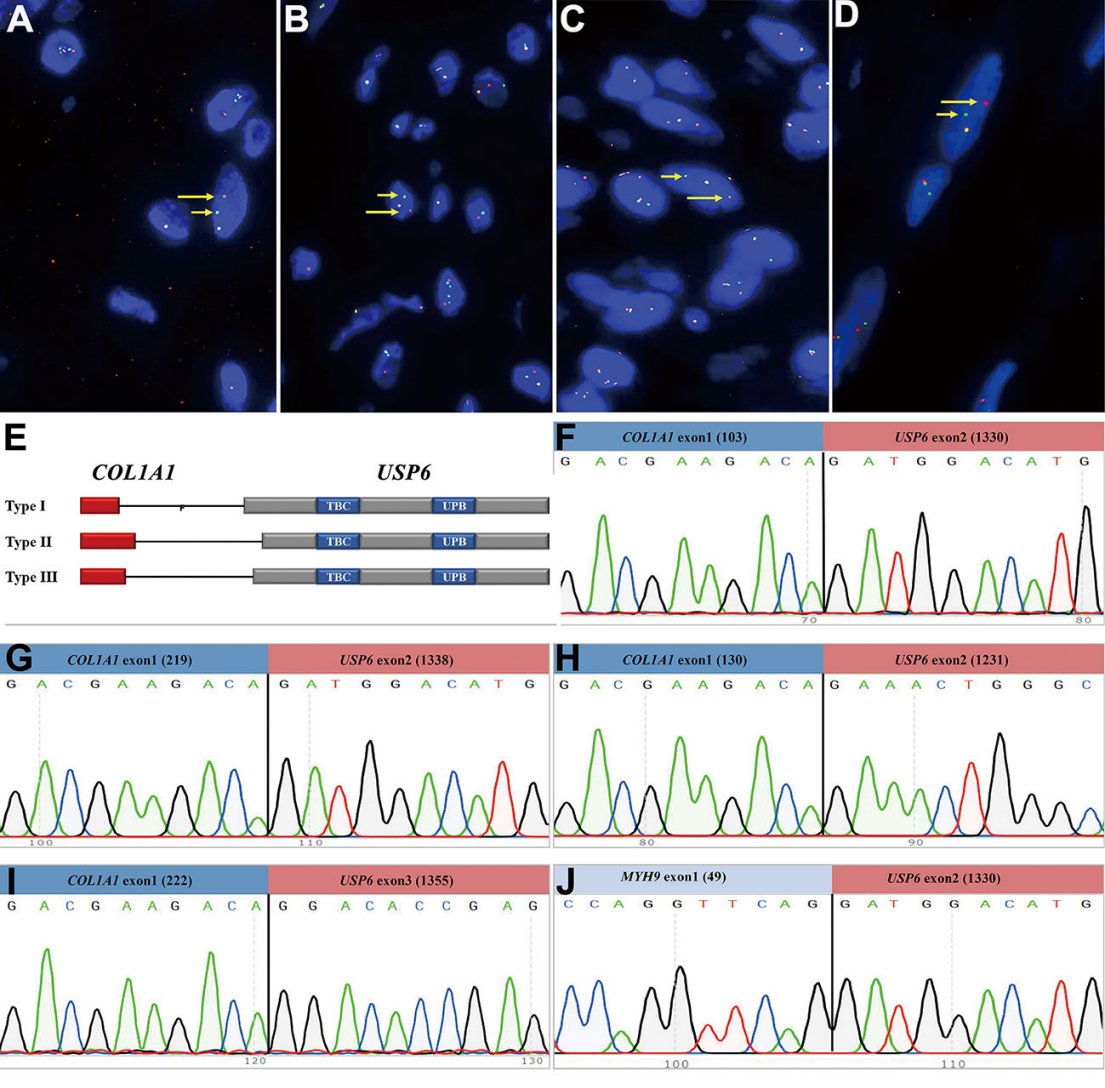
Findings of break-apart *USP6* fluorescence *in situ* hybridization and reverse transcription polymerase chain reaction for identifying common *USP6* fusion partners. **(A)** Break-apart *USP6* FISH showed split red (long arrow) and green (short arrow) signals in myositis ossificans (Case 42). **(B)** *USP6* FISH showed split red (long arrow) and green (short arrow) signals in fibro-osseous pseudotumor of digits (Case 49). **(C)** *USP6* FISH showed split red (long arrow) and green (short arrow) signals in soft tissue aneurysmal bone cyst (No. 60). **(D)** *USP6* FISH showed split red (long arrow) and green (short arrow) signals in fasciitis ossificans (No. 63). **(E)** Diagram showing the overall structure of *COL1A1::USP6* fusion genes. **(F)** One myositis ossificans (Case 22) showed the nucleotide (103, NM_000088) of *COL1A1* exon 1 fused with a nucleotide (1330, NM_0004505) of *USP6* exon 2 (type I). **(G)** One fasciitis ossificans (Case 71) showed the last nucleotide (219, NM_000088) of *COL1A1* exon 1 fused with a nucleotide (1338, NM_0004505) of *USP6* exon 2 (type II). **(H)** One myositis ossificans (Case 32) showed the last nucleotide (130, NM_000088) of *COL1A1* exon 1 fused with a nucleotide (1231, NM_0004505) of *USP6* exon 1 (type III). **(I)** One fasciitis ossificans (Case 63) showed a nucleotide (222, NM_000088) of *COL1A1* exon 1 fused with a nucleotide (1355, NM_0004505) of *USP6* exon 3. **(J)** One myositis ossificans (Case 42) showed the last nucleotide (49, NM_000088) of *MYH9* exon 1 fused with a nucleotide (1330, NM_0004505) of *USP6* exon 2.

**Figure 5:**
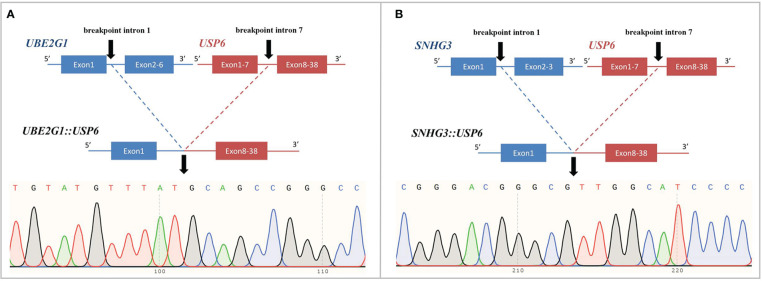
Novel fusion partners identified in *USP6*-associated soft tissue tumors with bone metaplasia. **(A)** NGS-based technology showing detection of *UBE2G1* (exon1)*:: USP6* (exon8-38) fusion and breakpoint information between the two genes. Sanger sequencing analysis confirmed the presence of *UBE2G1::USP6* fusion. **(B)** NGS-based technology showing detection of *SNHG3* (exon1)*::USP6* (exon8-38) fusion and breakpoint information between the two genes. Sanger sequencing analysis confirmed the presence of *SNHG3::USP6* fusion.

In current studies, FISH has been performed on tissues in 43 cases with available undecalcified tissues; 27 cases, including 17 MOs, 2 FOPDs, 2 ST-ABCs, and 6 FOs, were successfully detected, and 16 of them failed due to poor DNA quality or tissue falling. Among these cases with interpretable results, FISH showed rearrangements of *USP6* in 20%~60% of neoplastic cells in 22 cases (81.5%) (**Figures 4A–D**), and 5 cases were negative for *USP6* rearrangements (**Table 1**). The positive rates for *USP6* rearrangement among cases of MO, FOPD, ST-ABC and FO were 70.6% (12/17), 100.0% (2/2), 100% (2/2) and 100.0% (6/6), respectively.

**Table 1:** Genetic findings of *USP6*-associated soft tissue tumors with bone metaplasia.

Case No.	Diagnosis	Location	Sex	Age	Trauma History	Size (cm)	*USP6* FISH	RT-PCR and Sanger sequencing/ Next-generation-based sequencing
4	MO	Elbow	M	49	NA	2.0	+	*COL1A1 exon1::USP6 exon2*
7	MO	Hip	F	14	No	4.1	+	*COL1A1 exon1::USP6 exon2*
10	MO	Back	M	14	No	3.5	+	–
14	MO	Arm	F	62	NA	6.0	–	ND
18	MO	Hip	M	46	No	5.3	+	*COL1A1 exon1::USP6 exon2*
19	MO	Thigh	F	60	No	3.0	–	ND
20	MO	inguinal region	M	21	Yes	NA	–	ND
22	MO	Neck	M	5	No	6.0	+	*COL1A1 exon1::USP6 exon2*
30	MO	Hip	M	12	No	NA	+	–
32	MO	Thigh	M	22	NA	10.0	+	*COL1A1 exon1::USP6 exon1*
33	MO	Thigh	M	36	No	7.0	+	ND (poor RNA quality)
37	MO	Thigh	M	36	NA	3.7	–	ND
39	MO	Hip	M	7	No	3.0	+	*UBE2G1 exon1::USP6 exon 8-38*
41	MO	Thigh	F	54	No	3.5	–	ND
42	MO	Hip	M	15	No	5.0	+	*MYH9 exon1::USP6 exon2*
43	MO	Crus	M	14	No	4.0	+	ND (poor RNA quality)
44	MO	Thigh	F	15	No	4.0	+	ND (poor RNA quality)
49	FOPD	Finger	M	64	NA	0.8	+	ND (poor RNA quality)
58	FOPD	Finger	M	20	Yes	NA	+	*COL1A1 exon1::USP6 exon2*
60	ST-ABC	Hip	M	14	Yes	5.0	+	*COL1A1 exon1::USP6 exon1*
61	ST-ABC	Lumbar vertebral side	M	15	No	4.0	+	*COL1A1 exon1::USP6 exon2*
63	FO	Thigh	F	44	No	12.0	+	*COL1A1 exon1::USP6 exon3*
64	FO	Infraclavicularis	M	28	No	4.0	+	*COL1A1 exon1::USP6 exon2*
65	FO	Knee	M	10	No	5.0	+	*COL1A1 exon1::USP6 exon2*
66	FO	Arm	M	32	No	2.0	+	*SNHG3 exon1::USP6 exon 8-38*
71	FO	Knee	M	18	No	5.0	+	*COL1A1 exon1::USP6 exon2*
72	FO	Thigh	F	36	No	5.0	+	*COL1A1 exon1::USP6 exon2*

MO, myositis ossificans; FOPD, fibro-osseous pseudotumor of digits; ST-ABC, soft tissue aneurysmal bone cyst; FO, fasciitis ossificans; F, female; M, male; NA, not available; ND, not done; FISH, Fluorescence in situ hybridization; RT-PCR, Reverse Transcription-Polymerase Chain Reaction. +, positive; -, negative.

Among those 22 patients with positive *USP6* rearrangements, RT–PCR was successfully performed in 18 cases (3 MOs and 1 FOPD with poor RNA quality failed testing); 13 cases (72.2%), including 5 MOs, 1 FOPD, 2 ST-ABCs and 5 FOs, were positive for *COL1A1::USP6* fusion, and one case with MO was positive for *MYH9::USP6* fusion. For *COL1A1::USP6* fusion types (**Figure 4E**), 1 MO, 1 FOPD and 3 FO cases were found with fusion transcript type 1 (nucleotide 103, NM_000088 exon 1; nucleotide 1330, NM_0004505 exon 2) (**Figure 4F**), 3 MO, 1 ST-ABC and 1 FO cases were found with fusion type II (nucleotide 219, NM_000088 exon 1; nucleotide 1338, NM_0004505 exon 2) (**Figure 4G**), and 1 MO and 1 ST-ABC demonstrated fusion transcript type III (nucleotide 130 exon 1, NM_000088; nucleotide 1231, NM_0004505 exon 1) (**Figure 4H**). One FO case was found with a special transcript type with *COL1A1* (nucleotide 222, NM_000088 exon 1) and *USP6* (nucleotide 1355, NM_0004505 exon 3) fusion (**Figure 4I**). Additionally, one MO case showed fusion of exon 1 (nucleotide 49, NM_000088) of *USP6* and exon 2 (nucleotide 1330, NM_0004505) of *MYH9* instead of any transcript type of *COL1A1*::*USP6* fusion (**Figure 4J**).

**Figure 6:**
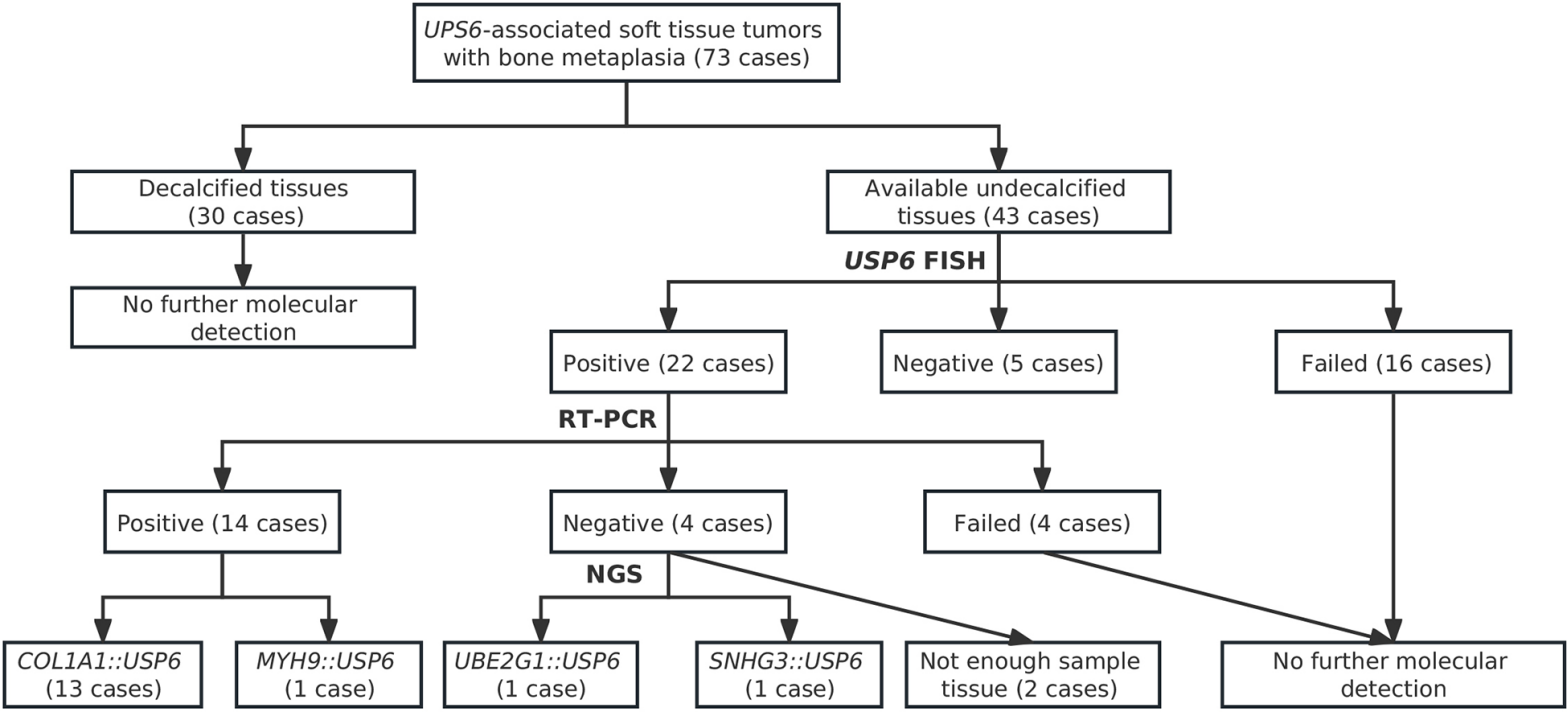
Summary of process and results for genetic testing of *USP6*-associated tumors.

Four *USP6-*rearranged lesions were negative for *COL1A1::USP6*, and only two of them (1 MO and 1 FO) underwent next-generation-based sequencing technology, while the tissues of the other two cases were not sufficient to perform a further study. A novel fusion of *UBE2G1* (exon 1, NM_003342) and *USP6* (exon 8-38, NM_001304284) was identified in one MO case (Case 39) (**Figure 5A**), while a novel fusion of *SNHG3* (exon 1, NR_002909) and *USP6* (exon 8-38, NM_001304284) was demonstrated in one FO case (Case 66) (**Figure 5B**). The presence of the fusion at the DNA level was further validated by RT–PCR and Sanger sequencing using corresponding primers (*UBE2G1-F*: 5’-AGGCTGGTCTTGAACTCCTGA-3’and *USP6-R*: 5’-CGTGTGTGTTGCTTCTCTGGC-3’; *SNHG3-F*: 5’-TCTTAGTGGAGACGGGGTTTC-3’ and *USP6-R*: 5’-AGCTAGAGGATCATGTGCGGA-3’). The process and results of genetic testing are summarized in **Figure 6**.

### Treatment and follow-up

Treatment information was obtained in 63 cases. Sixteen patients underwent biopsy, 41 patients underwent local complete resection, 3 patients underwent wide resection, 2 patients underwent partial mass resection, and 1 patient underwent curettage. Follow-up information was obtained for 36 patients (4 patients underwent biopsy, and 32 patients underwent resection), and the follow-up times ranged from 1 to 127 months (median, 26 months). Four patients survived with tumors after the biopsy without tumor progression. Twenty-nine cases showed no tumor progression after biopsy or surgery, and 5 cases (5/34, 14.7%) experienced postoperative recurrence, including 1 MO with curettage, 2 FOPDs with partial mass resection, 1 MO and 1 FO with complete resection (**Table 2**).

**Table 2:** Clinicopathologic and genetic results of 5 recurrent cases.

Case No.	Final Diagnosis	Sex	Age	Truma History	Duration time (mo)	Location	Size (cm)	Depth	Histological morphology	mitosis	Case source	Referal diagnosis	Genetic findings	Treatment	Recurrence (mo)
3	MO	M	17	Yes	12	Elbow	6.0	Intramuscular	zonal	4/10HPF	Consultation cases/In-house case	Callus?	ND (decalcification)	Curettage	11
34	MO	F	13	No	8	Thigh	10.0	Intramuscular	zonal	6/10HPF	In-house case	MO?	ND (decalcification)	Local complete resection	24
55	FOPD	F	12	No	12	Toe	3.0	Subcutaneous	No zonal	0/10HPF	In-house case	osteochondroma?	ND (decalcification)	Partial resection	NA
59	FOPD	M	16	No	6	Finger	3.0	Subcutaneous	No zonal	0/10HPF	In-house case	FOPD	ND (decalcification)	Partial resection	6
63	FO	F	44	No	2	Thigh	12.0	Subcutaneous	Zonal, NF-like morphology	8/10HPF	Consultation cases/In-house case	FO? Osteosarcoma?	*COL1A1exon1::USP6 exon3*	Local complete resection	2

MO, myositis ossificans; FOPD, fibro-osseous pseudotumor of digits; FO, fasciitis ossificans; F, female; M, male; NF, nodular fasciitis; ND, not done; NA, not available.

Details of the clinicopathological and genetic characteristics of different subtypes of *USP6*-associated soft tissue tumors with bone metaplasia are shown in **Supplemental Table 2**.

## Discussion

*USP6*-associated soft tissue tumors with bone metaplasia are a subgroup of myofibroblasts/fibroblastic proliferative lesions with metaplastic osteoid components, mainly including MO, FOPD, ST-ABC and FO. This group of neoplasms is not clinically common, but its histology shows pseudosarcomatous changes with the formation of an osteoid component, making it susceptible to misdiagnosis as a malignancy.

In our cohort, there were 73 cases of *USP6*-associated soft tissue tumors with bone metaplasia, including 44 MOs, 15 FOPDs, 2 ST-ABCs and 12 FOs. As we know, this is one of the largest groups for exploring the clinicopathologic and molecular characteristics of this entity. The diagnosis age in this group was wide, ranging from 2 to 80 years old (median: 31 years). Consistent with previous reports, these tumors can occur in all age groups, but they are more common in young adults between 20~40 years old (5, 16, 17). Fourteen patients (14/48, 29.2%) had a clear history of trauma, but the history of repeated minor trauma might be neglected. In this group, except for FOPD, the lesion sites of the other subtypes were mainly in the lower limbs. The predisposing site of our group was different from that of traditional NF, which more often involves the upper limbs and trunk (18). Notably, FO is currently classified as a special subtype of NF. However, according to the results of our study, we found that the most common site of FO was the lower limbs (9/12, 75%) instead of the upper limbs and trunk. Three FOs were also reported in the latest study, of which 2 cases were identified in the lower extremities, which was consistent with our findings (5). As for imaging examinations in our study, the results were consistent with those reported in previous literature (5, 9, 10, 16, 19).

Histologically, this group showed hyperplastic myofibroblasts/fibroblasts and osteoid tissues with different degrees of maturity. The histological morphology of MO/FOPD and ST-ABC in the present study were essentially consistent with the description in the literature (16, 17, 20). There have been few case reports of FO, and the deposition pattern of osteoid tissues is mainly described as having a haphazard distribution similar to that of FOPD. However, MO-like zonal deposition pattern was observed in 50% of FO cases in our group, which indicated that there was evident histological overlap between FO and the other three subtypes of *USP6*-associated soft tissue tumors with bone metaplasia.

**Figure 7:**
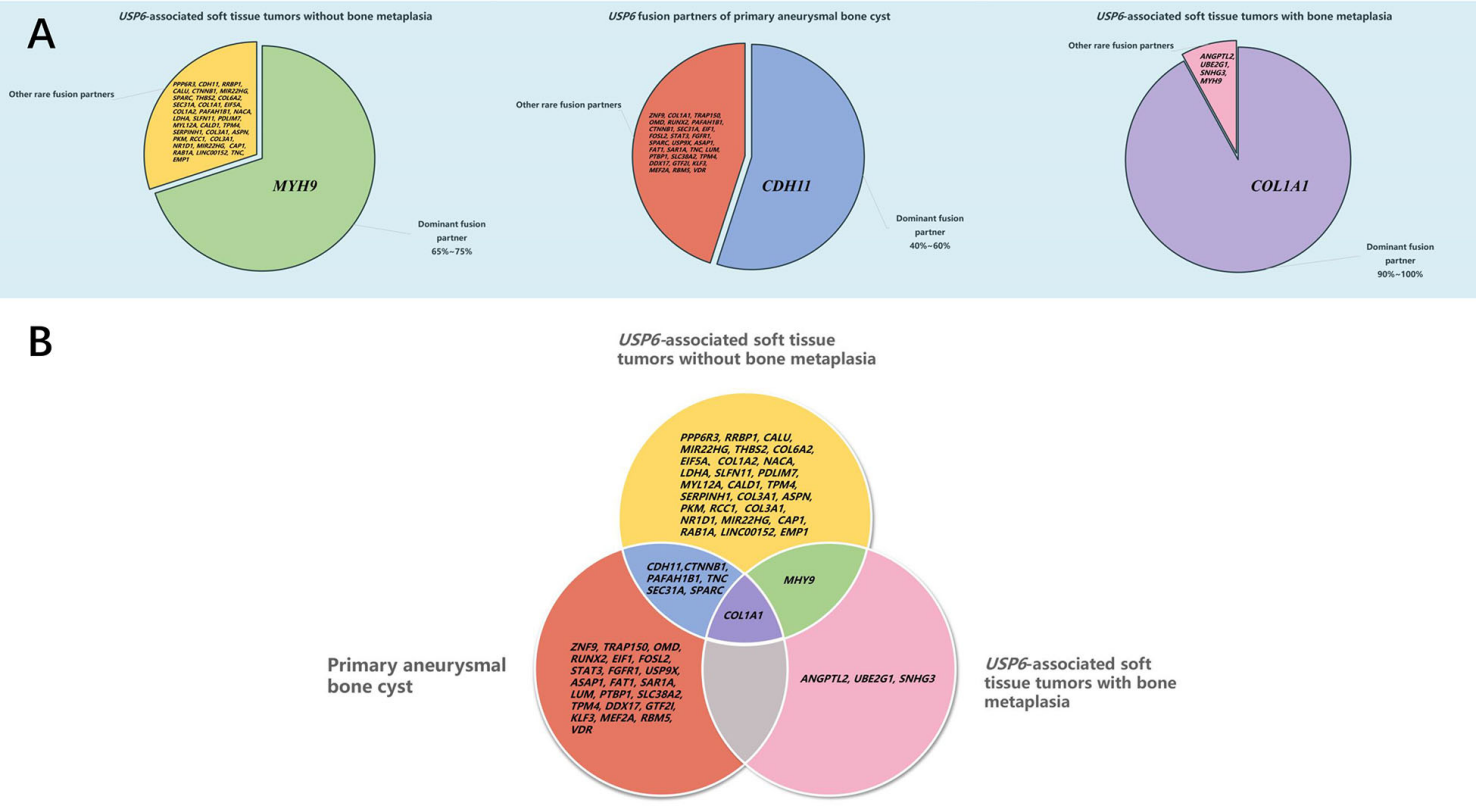
Distribution of *USP6* fusion partners in *USP6*-associated tumors. **(A)** Distribution of the *USP6* fusion gene in different *USP6*-associated tumors. **(B)** Overlap of the *USP6* fusion gene in different *USP6*-associated tumors.

At present, soft tissue tumors in osteoid tissues have been successively confirmed to be *USP6*-associated tumors. Studies have suggested that the positive rate of *USP6* rearrangement/fusion in MO/FOPD, ST-ABC and FO ranges from 16.7% to 100% (5, 8–10, 17, 19, 21). However, it should be emphasized that the overall sample size of existing studies is relatively small, with only 4 studies with more than 10 cases (11 cases (8), 12 cases (10), 12 cases (21), 14 cases (5)). Our study had the largest number of cases in a single center, and the positive rate of *USP6* rearrangement detected by FISH in this group of cases was 81.5% (22/27). In recent years, an increasing number of studies have focused on the fusion partners of these tumors and the relationship between them. Our study suggested that *COL1A1* (13/18, 72.2%) was the most frequent fusion partner in MO/FOPD, ST-ABC and FO, which was consistent with previous studies (67.7%~100%) and differed from classical primary ABC and NF (5, 10). Notably, despite the presence of significant ossification, FO is currently classified as a variant of NF. Although it has also been reported that *COL1A1* was found to be the fusion partner in a few NFs (22), *COL1A1* was the unique *USP6* fusion partner of FO in this study, which was consistent with the findings of Wang and colleagues (5). Meanwhile, we found that the most common site of FO was the lower limbs instead of the upper limbs of NF, suggesting that FO seemed to be more closely related to MO/FOPD and ST-ABC. Notably, in *USP6*-associated neoplasms, although an increasing number of partner genes have been identified, there is a preference for these partner genes in different tumor subtypes (5–7, 10, 14, 15, 17, 19, 23–35) (**Figure 7A**). Intriguingly, according to our study and previous literature, almost all of the *USP6*-associated neoplasms with bone metaplasia adopt *COL1A1* as the fusion partner, including MO, FOPD, ST-ABC and FO (5, 10, 17, 19, 25). As reported, *COL1A1* encodes the pro-alpha1 chain of type I collagen, which is associated with osteogenesis imperfecta and osteoporosis (36). It should be noted that the fusion of *COL1A1* and *USP6* still retained the whole *USP6* coding sequence of the open reading frame, namely, the fusion of the partner gene exon1 and the *USP6* gene exon1 or/and exon2. Although this fusion is expected to lead to the high expression of *USP6* or be the main mechanism of *USP6* gene-mediated tumor pathogenesis, the high percentage of *COL1A1* as a fusion partner in these tumor subtypes still likely has a potential association with the osteoid formation on the histology, but further research is needed. In addition, individual *USP6* fusion partners that overlap between these tumor subtypes have been identified (3, 7, 11, 15, 22, 35–39) (**Figure 7B**). Although such cases are limited, the possibility of some crossover or even transition between *USP6*-associated neoplasms cannot be ruled out.

Notably, although *COL1A1* was the most frequent fusion partner in this entity, we still identified *MYH9::USP6* fusion in one case of MO (Case 42). At present, after thoroughly reviewing the English literature, *MHY9* has not been reported as a *USP6* fusion partner in MO/FOPD, ST-ABC and FO. As reported, *MYH9::USP6* fusion is the most frequent fusion type in NF (including variants of NF) and FTS in *USP6*-associated tumors (4, 5, 40). After carefully reviewing the slides of this lesion, we found that it was an intramuscular mass and demonstrated a prominent zonation pattern in focal areas (**Figure 2D**) and obvious NF-like morphology in some foci (**Figure 2C**), which indicated there are some overlapping histological features as well as molecular findings among different subtypes of *USP6*-associated neoplasms, for example, as the major fusion type in the primary ABC, *CDH11::USP6* also can be detected in the NF, but how they work in building the bridge between them are still not clear and needs to be further studied in a larger cohort.

In this study, we also identified a novel ubiquitin-conjugating enzyme E2 G1 *(UBE2G1)::USP6* gene fusion in one case of MO (Case 39) and a novel small nuclear protein RNA host gene 3 (*SNHG3)::USP6* fusion in one case of FO (Case 66). To our knowledge, fusions of these two genes with *USP6* have not been previously reported. *UBE2G1* is located on chromosome 17p13.2; it encodes a member of the E2 ubiquitin-conjugating enzyme family and catalyzes the covalent attachment of ubiquitin to other proteins (41). *SNHG3* is located on chromosome 1p35.3, which belongs to a group of long noncoding RNAs associated with multiple cancers and is dysregulated in multiple cancers (42). Recent studies have shown that *SNHG3* expression is higher in many tumors, such as osteosarcoma, breast cancer and hepatocellular carcinoma (42). Similar to previous findings (4, 43), the entire coding region of *USP6* was preserved in these two rearrangements, which likely leads to activat*ing USP6 t*ranscription and subsequent neoplastic processes.

Notably, except for *MYH9*, *UBE2G1* and *SNHG3*, which were discovered in our study, the unusual *USP6* partner *ANGPTL2* has also been identified in one MO-like ST-ABC (17). However, despite the presence of uncommon fusion types, no peculiar clinicopathologic findings were identified in these 4 cases. Whether the novel fusions may be related to the clinicopathological features of these tumors remains to be further investigated. Additionally, according to many reported studies, in most cases of *USP6*-associated tumors, the fusion site of *USP6* was exon 1 or/and exon 2 (4, 5, 10). In the current study, we found rare fusion sites of the *USP6* gene, which were exon 3 and exon 8 of *USP6* (NM_001304284). In addition, a previous study by our team also reported an equally rare case of atypical NF in children at the fusion site of exon 9 of *USP6* (NM_001304284) (15). However, whether these rare fusion sites are related to clinicopathologic characteristics still needs further study.

**Figure 8:**
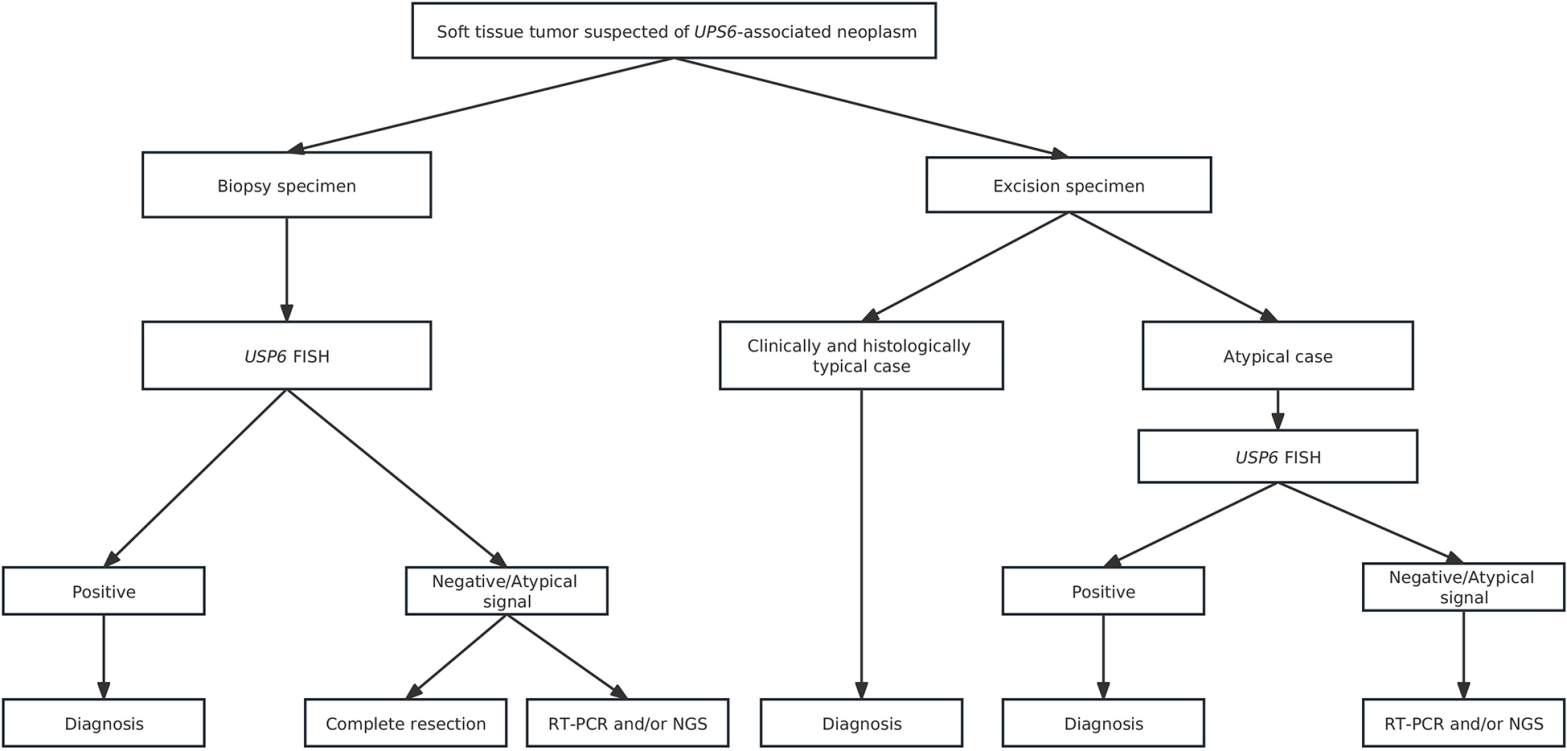
Diagnostic algorithm for soft tissue tumors suspected of *UPS6*-associated neoplasms.

For differential diagnosis, *USP6*-associated soft tissue tumors with bone metaplasia are extremely easily confounded with extraskeletal osteosarcoma, especially in MO cases. Ten of 33 consultation cases were initially considered extraskeletal osteosarcoma in the local hospital. Clinically, extraskeletal osteosarcoma more commonly occurs in middle-aged and elderly populations and often lacks an injury history. Histologically, osteoid components of extraskeletal osteosarcoma are often arranged in a reverse zonation pattern. Obvious cell atypia with pathologic mitosis can be present among neoplastic cells (44). In addition, *USP6*-associated soft tissue tumors with bone metaplasia need to be differentiated from other osteogenic sarcomas, such as malignant peripheral nerve sheath tumors with heterogeneous bone differentiation and dedifferentiated liposarcomas with heterogeneous bone differentiation. Malignant peripheral nerve sheath tumors is usually a rare, high-grade sarcoma with high morphological heterogeneity. The tumor cells are long and fusiform with wavy, curved, comma-shaped or asymmetrically ovoid nuclei, the chromatin is usually uniform or dense, and pathological mitosis is easy to observe. Complete loss of H3K27me3 along with *SUZ12* and *EED* gene deletions are frequently seen in this entity (45), which are invaluable in the diagnosis of challenging cases. For dedifferentiated liposarcomas with heterogeneous bone differentiation, except for dedifferentiated bone elements, well-differentiated liposarcomatous components are characteristically present. More importantly, consistent *MDM2* and/or *CDK4* amplification is present in dedifferentiated liposarcomas (46), which is absent in *USP6*-associated soft tissue tumors with bone metaplasia. In addition to those malignant tumors, *USP6*-associated soft tissue tumors with bone metaplasia should also be differentiated from some benign lesions, including proliferative fasciitis/proliferative myositis, bizarre parosteal ostochondromatous proliferation (Nora’s lesion) and subungual exostosis. Proliferative fasciitis/proliferative myositis is a type of myofibroblast/fibroblast proliferative disease that occurs in the subcutaneous fascia and muscle. It is characterized by a scattered distribution of ganglion-like cells, interstitial mucinous degeneration and collagenization in the background (47). In addition, no *USP6* rearrangement was identified in proliferative fasciitis/proliferative myositis, indicating that proliferative fasciitis/proliferative myositis is a real reparative lesion instead of a neoplastic change. Nora’s lesion often presents bizarre, enlarged nuclei and stroma with a characteristically basophilic tinctorial quality. Additionally, genetic changes with t(1;17) (q32; q21) and inv (7) and inv (6) were recurrently identified in Nora’s lesion and were not commonly seen in FOPD, MO and FO (48). FOPD should also be distinguished from subungual exostosis, as it is more prone to involve digits. Unlike FOPD, subungual exostosis commonly presents as a lesion with an irregular bone-cartilage interface with enlarged, atypical and binucleated chondrocytes histologically and harbors a t(X;6) (q24-q26; q15-q25) change genetically (49). In clinical practice, molecular testing is usually unnecessary for clinically and histologically typical cases. However, in challenging cases, *USP6* FISH, RT-PCR and/or NGS may be used to confirm the diagnosis. We recommend the diagnostic algorithm shown in the figure to reduce misdiagnosis as much as possible (**Figure 8**).

*USP6*-associated soft tissue tumors with bone metaplasia are benign lesions. Currently, local resection is the most commonly used and most effective treatment. Postoperative recurrence is rare (16). Patients with incomplete resection are likely to experience recurrence. In our study, among the patients with follow-up information, 4 patients who received biopsy only survived with tumors without tumor progression; most of the patients showed no evidence for recurrence, while 5 patients (5/32, 15.6%), including 2 MOs, 2 FOPDs and 1 FO, showed recurrence after surgery. Those two FOPD patients underwent partial resection with positive margins to retain the normal function of the finger or toe, and one MO was not completely curetted, which may lead to relapses of these tumors. Another MO and FO with recurrence underwent complete local resection and showed a large tumor size of over 10.0 cm (10.0 cm and 12.0 cm). In this study, the tumor volumes of 2 cases with recurrence after complete resection were large (over 10.0 cm), among which one case also harbored a rare fusion site (Case 63) (the other case did not undergo genetic testing due to sample decalcification). Notably, the FO recurrent case (Case 63) had special histological morphology. Some areas of the tumor showed invasive growth, with the active proliferation of myofibroblasts/fibroblasts, messy distribution of osteoid tissue, and reverse distribution of focal areas similar to osteosarcoma (**Figures 3B–D**). Due to the large size of the tumor, osteosarcoma could not be excluded from the original diagnosis after the lesion recurrence, and the diagnosis of FO was finally confirmed by *USP6* FISH test. However, whether these were related to clinical conditions (especially biological behavior) still needs to be further studied by increasing the number of cases. Given the nature of this group of tumors, if the lesion can be diagnosed in the biopsy, it may provide more treatment options for patients. However, due to the small size of biopsies, it is difficult to diagnose these tumors only by histology and immunohistochemistry. FISH detection of *USP6* rearrangement will be valuable for clarifying the diagnosis, and the method of surgery can be determined according to the tumor size, location, and follow-up.

## Conclusions

In summary, *USP6*-associated soft tissue tumors with bone metaplasia include MO, FOPD, ST-ABC and FO. Here, we explored the peculiar clinicopathologic and molecular features of this entity in one of the largest cohorts. Tumors can occur in all age groups but most often affect young adults. Different subtypes of this entity not only share overlapping clinicopathological features but also exhibit similar genetic changes, namely, consistent *USP6* rearrangement and frequent *COL1A1::USP6* fusion. Notably, for FO, the lower limbs are commonly involved sites, and *COL1A1* is the most frequent fusion partner, suggesting that FO may be closer to MO/FOPD and ST-ABC than conventional NF. Another point we should address is that *MYH9::USP6* was first identified in MO, and the novel *USP6* fusion partners *UBE2G1* and *SNHG3* were uncovered in one case with MO and one case with FO, respectively, expanding our knowledge of *USP6*-associated soft tissue tumors with bone metaplasia. The prognosis of this entity is good, and local complete resection is the most effective treatment. Recurrence may be associated with incomplete resection and/or large tumor size (over 10.0 cm), and whether rare fusion sites or novel/uncommon fusion partners are correlated with clinical parameters still needs to be further studied in larger cohorts.

## Data availability statement

The raw data supporting the conclusions of this article will be made available by the authors, without undue reservation.

## Ethics statement

This study was approved by the Ethics Committee on Biomedical Research, West China Hospital of Sichuan University (No. 793, 2021). Approval for waiver of informed consent was obtained for this study.

## Author contributions

YZ: collected the clinicopathological data, performed the histopathological examinations, and molecular detection, and prepared the manuscript. YQ: analyzed the molecular data and prepared the manuscript. XZ: performed the histopathological examinations. XH and CC: helped with data review. MC: helped with molecular experiments. HZ: the corresponding author, was responsible for study design, histopathological and molecular examinations, and manuscript revision. All authors contributed to the article and approved the submitted version.
