# Supplementary material for: USP6-associated soft tissue tumors with bone metaplasia: Clinicopathologic and genetic analysis and the identification of novel USP6 fusion partners
Source: Front Oncol. 2023 Jan 16;12:1065071. doi: 10.3389/fonc.2022.1065071 (PMC9885078; doi:10.3389/fonc.2022.1065071)
Supplement: Supplementary file 1 [file Table_1.docx]

**Supplemental Table 1. Primer sequences for reverse transcription-polymerase chain reaction (RT-PCR) detection**

| **Fusion types** | [**Forward**](javascript:;) [**primer**](javascript:;) | **Reverse primer** |
| --- | --- | --- |
| ***PPP6R3::USP6*** | *PPP6R3-*Forward：  5'-TGCTTGATACGTCCGCCATTTTGGG-3'  *PPP6R3-*Forward：  5'-TGCTTGATACGTCCGCCATTTTGGG-3'  *PPP6R3-*Forward：  5'-TGCTTGATACGTCCGCCATTTTGGG-3' | *USP6*-Reverse-1：  5'-GTACGATCGGCCTCCTGGGATGTTC-3'  *USP6*-Reverse-2：  5'-GATGTGGATGTGAACTGCGGTCAGG-3'  *USP6*-Reverse-3：  5'-GAATTTTCTTCGCCTCCCGTGCAGT-3' |
| ***MYH9::USP6*** | *MYH9*-Forward-1：  5'-ACGGAAGGCTAAGCAAGGCTG-3'  *MYH9*-Forward-2：  5'-GCACGGAAGGCTAAGCAAG-3′  *MYH9*-Forward-2：  5'-GCACGGAAGGCTAAGCAAG-3′  *MYH9*-Forward-3：  5'-GGGGCAGATCCAGGTTCAG-3'  *MYH9*-Forward-3：  5'-GGGGCAGATCCAGGTTCAG-3'  *MYH9*-Forward-4：  5'-GAAGGCTAAGCAAGGCTGAC-3'  *MYH9*-Forward-5：  5'-TGCTGCAGCTCCCGCCTCGTGC-3'  *MYH9*-Forward-6：  5'-ATCACCGCGGTTCCTG -3'  *MYH9*-Forward-6：  5'-ATCACCGCGGTTCCTG -3'  *MYH9*-Forward-7：  5'-ACGGAAGGCTAAGCAAGGCTG -3'  *MYH9*-Forward-7：  5'-ACGGAAGGCTAAGCAAGGCTG -3' | *USP6*-Reverse-4：  5'-TAGTTTGCAGGCACAGGAGCGGAA-3'  *USP6*-RS：  5'-GGATGTGGATGTGAACTGCG-3′  *USP6*-RL：  5'-CGGTGTCCCTTGTCATACTTC-3′  *USP6*-Reverse-5：  5'-GAAACTGGGCATCTCTGTGGC-3'  *USP6*-Reverse-6：  5'-GATGGACATGGTAGAGAATGC-3'  *USP6*-Reverse-7：  5'-GTGAAGAGCCTGTGATGGTCTATT-3'  *USP6*-Reverse-8：  5'-ACGGGCTCAGGCCCCTTGTCCT-3'  *USP6*-Reverse-9：  5'-TACGATCGGCCTCCTGGGATG -3'  *USP6*-Reverse-10：  5'-CTTCCGCTCCTGTGCCTGCAAACTA -3'  *USP6*-Reverse-9：  5'-TACGATCGGCCTCCTGGGATG -3'  *USP6*-Reverse-10：  5'-CTTCCGCTCCTGTGCCTGCAAACTA -3' |
| ***COL1A1::USP6*** | *COL1A1*-77F：  5'-CCCCTCCCCAGCCACAAAGAGT-3'  *COL1A1*-77F：  5'-CCCCTCCCCAGCCACAAAGAGT-3'  *COL1A1*-37F：  5'-CCCAGCCACAAAGAGTCTACAT-3'  *COL1A1*-37F：  5'-CCCAGCCACAAAGAGTCTACAT-3'  *COL1A1*-4F：  5'-AGACATGTTCAGCTTTGTGGAC-3'  *COL1A1*-4F：  5'-AGACATGTTCAGCTTTGTGGAC-3'  *COL1A1*-Forward-1：  5'-TCGAGGGCCAAGACGAAGA-3'  *COL1A1*-Forward-2：  5'-CTAGGGTCTAGACATGTTCAGCTTT-3'  *COL1A1*-Forward-3：  5'-TCTAGGGTCTAGACATGTTCAGCTT-3'  *COL1A1*-Forward-4：  5'-AAGAGGAAGGCCAAGTCGAG-3' | *USP6*-2951R：  5'-GGGTACGTGTCTTCCCGGACAGC-3′  *USP6*-1863R：  5'-CCCGTGCAGTCACAGGAGGCA-3'  *USP6*+1781R：  5'-CTCGGTGTCCCTTGTCATACTT-3'  *USP6*+1747R：  5'-CTTCCGCTCCTGTGCCTGCAAACTA-3'  *USP6*+1781R：  5'-CTCGGTGTCCCTTGTCATACTT-3'  *USP6*+1747R：  5'-CTTCCGCTCCTGTGCCTGCAAACTA-3'  *USP6*-Reverse-7：  5'-AGTATGTCCTTCCGCTCCTGT-3'  *USP6*-Reverse-11：  5'-GATGGTCTATTCCAGGGATGTG-3'  *USP6*+1781R：  5'-CTCGGTGTCCCTTGTCATACTT-3'  *USP6*-Reverse-12：  5'-AAATCGTCTACATGGGCAGTG-3' |
| ***CDH11::USP6*** | *CDH11*+71F：  5'-CGCCGCTGACTTGTGAAT-3'  *CDH11*+83F：  5'-GTGAATGGGACCGGGACT-3'  *CDH11-*Forward：  5'-CGGCTCGGAGGTTGCGTCC-3'  *CDH11-*Forward：  5'-CGGCTCGGAGGTTGCGTCC-3'  *CDH11*+83F：  5'-GTGAATGGGACCGGGACT-3' | *USP6*+1781R：  5'-CTCGGTGTCCCTTGTCATACTT-3'  *USP6*+1781R：  5'-CTCGGTGTCCCTTGTCATACTT-3'  *USP6*_Ex2_R：  5'-CAGCCCAGCTCGGTGTCCC-3'  *USP6*_Ex1_R：  5'-CCTGGGATGTTCAGGGCCAC-3'  *USP6*+1736R：  5'-CAGGAGCGGAAGGACATACTTA-3' |
| ***SEC31A::USP6*** | *SEC31A*-Forward-1：  5' -ATGCTGGGAGAGTCCGACGAG-3' | *USP6*-Reverse-13：  5'-TACGATCGGCCTCCTGGGATGT-3' |
| ***RUNX2::USP6*** | *RUNX2*-Forward-1：  5'-CAGTATTTACAACAGAGGGTAC-3'  *RUNX2*-Forward-1：  5'-CAGTATTTACAACAGAGGGTAC-3' | *USP6*_Ex2_R：  5'-CAGCCCAGCTCGGTGTCCC-3'  *USP6*_Ex1_R：  5'-CCTGGGATGTTCAGGGCCAC-3' |
| ***PAFA1B1::USP6*** | *PAFA1B1*-Forward-1：  5′-GGGAGTGAAGGACGGAAGAG-3′  *PAFA1B1*-Forward-2：  5'-CGGTGGATGGGAGTGAAGG-3' | *USP6*-Reverse-14：  5′-GGTGAAGAGCCTGTGATGGT-3′  *USP6*_Ex2_R：  5'-CAGCCCAGCTCGGTGTCCC-3' |
| ***COL1A2::USP6*** | *COL1A2*-Forward-1：  5'-GTGGATACGCGGACTTTGTT-3' | *USP6*-Reverse-15：  5'-AACGATCAATGCTGCTGTTG-3' |
