# Supplementary material for: USP6-associated soft tissue tumors with bone metaplasia: Clinicopathologic and genetic analysis and the identification of novel USP6 fusion partners
Source: Front Oncol. 2023 Jan 16;12:1065071. doi: 10.3389/fonc.2022.1065071 (PMC9885078; doi:10.3389/fonc.2022.1065071)
Supplement: Supplementary file 2 [file Table_2.docx]

**Supplemental Table 2. Clinicopathologic findings of *USP6-*associated soft tissue tumors with osteoid tissue**

| Case No. | Final diagnosis | Sex | Age | Trauma history | Duration time（mo） | Symptoms | Location | Size  (cm) | Follow-up（mo） | Zone distribution | NF-like morphology | Immunohistochemistry | mitosis | Case source | Referal diagnosis | FISH | RT-PCR/ NGS-based technology | Treatment |
| --- | --- | --- | --- | --- | --- | --- | --- | --- | --- | --- | --- | --- | --- | --- | --- | --- | --- | --- |
| 1 | MO | F | 50 | No | 1 | Painful and swelling | [Thigh](javascript:;) | 5.0 | NED/30 | No | Not [obvious](javascript:;) | ND | 2/10HPF | In-house case | MO？ | ND (decalcification) | ND | Local complete resection |
| 2 | MO | M | 48 | NA | 0.7 | Painless mass | abdominal wall | 7.5 | NA | Yes | [Obvious](javascript:;) | ND | 2/10HPF | Consultation cases | Osteosarcomas are not excluded | ND (tissue unavailable) | ND | Local complete resection |
| 3 | MO | M | 17 | Yes  (injury) | 12 | Painful and swelling | Elbow | 6.0 | [Recurrence](javascript:;) /123 | Yes | Not [obvious](javascript:;) | ND | 4/10HPF | Consultation cases | Callus？ | ND (decalcification) | ND | Curettage |
| 4 | MO | M | 49 | NA | 1 | NA | Elbow | 2.0 | NA | No | Not [obvious](javascript:;) | SMA（+）、MSA（-）、S-100（-）、Ki-67 <5%（+） | 6/10HPF | Consultation cases | Chondrogenic tumor？ | + | *COL1A1 exon1::USP6 exon2* | NA |
| 5 | MO | F | 46 | Yes (sports injury) | 0.5 | Painful mass | Waist | NA | NA | Yes | Not [obvious](javascript:;) | ND | 2/10HPF | Consultation cases | Osteosarcomas？ | Failed | ND | NA |
| 6 | MO | F | 57 | NA | 0.3 | Painful mass | [Thigh](javascript:;) | 5.0 | NA | Yes | Not [obvious](javascript:;) | SMA（+）、Desmin（-）、S-100（-）、β-catenin（-）、Ki-67 3～10%（+） | 2/10HPF | Consultation cases | Invasive spindle cell tumor | ND (tissue unavailable) | ND | Local complete resection |
| 7 | MO | F | 14 | No | 0.5 | Painful mass | Hip | 4.1 | NA | Yes | Not [obvious](javascript:;) | SMA（+）、Desmin（-）、EMA（-）、S-100（-）、Ki-67 3～10%（+） | 7/10HPF | Consultation cases | Extraskeletal osteosarcoma？ | + | *COL1A1 exon1::USP6 exon2* | Biopsy |
| 8 | MO | F | 48 | NA | NA | NA | Knee | 4.0 | NA | Yes | Not [obvious](javascript:;) | SMA（+）、Desmin（-）、S-100（-）、β-catenin（-）、Ki-67 1～2%（+） | 2/10HPF | Consultation cases | Osteosarcomas are not excluded | ND (tissue unavailable) | ND | Local complete resection |
| 9 | MO | M | 48 | NA | 0.6 | Painful mass | cubitus | 5.1 | NA | Yes | Not [obvious](javascript:;) | ND | 0/10HPF | Consultation cases | MO | Failed | ND | Local complete resection |
| 10 | MO | M | 14 | No | 0.3 | Painful and swelling | Back | 3.5 | NED/87 | Yes | Not [obvious](javascript:;) | ND | 5/10HPF | Consultation cases | Extraskeletal osteosarcoma？ | + | - | Local complete resection |
| 11 | MO | F | 37 | No | 8 | Painless mass | [Thigh](javascript:;) | 2.0 | NED/83 | Yes | Not [obvious](javascript:;) | ND | 3/10HPF | Consultation cases | NA | ND (tissue unavailable) | ND | Local complete resection |
| 12 | MO | F | 23 | NA | 0.7 | Painful mass | [Thigh](javascript:;) | NA | NA | Yes | Not [obvious](javascript:;) | SMA（+）、MSA（+）、Desmin（-）、S-100（-）、Ki-67 8%（+） | 5/10HPF | Consultation cases | MO | Failed | ND | NA |
| 13 | MO | F | 27 | NA | NA | NA | [Thigh](javascript:;) | NA | NA | Yes | Not [obvious](javascript:;) | ND | 4/10HPF | Consultation cases | NA | ND (tissue unavailable) | ND | NA |
| 14 | MO | F | 62 | NA | 0.3 | Painful mass | Arm | 6.0 | NA | Yes | Not [obvious](javascript:;) | ND | 3/10HPF | Consultation cases | NA | - | ND | Biopsy |
| 15 | MO | M | 36 | NA | NA | NA | Chest wall | 5.0 | NA | No | [Obvious](javascript:;) | SMA（+）、β-catenin（-）、Desmin（-）、Ki67 30%（+） | 8/10HFP | Consultation cases | Spindle cell tumor | ND (tissue unavailable) | ND | NA |
| 16 | MO | M | 54 | Yes (sports injury) | 0.7 | [Pruritus](javascript:;) | Cubitus | 2.5 | NED/12 | No | [Obvious](javascript:;) | ND | 0/10HPF | Consultation cases | NA | ND (tissue unavailable) | ND | NA |
| 17 | MO | F | 34 | Yes (sprain) | 3 | Painful mass | [Thigh](javascript:;) | NA | NED/29 | Yes | [Obvious](javascript:;) | ND | 3/10HPF | Consultation cases | MO | ND (tissue unavailable) | ND | NA |
| 18 | MO | M | 46 | No | NA | NA | Hip | 5.3 | NA | No | Not [obvious](javascript:;) | ND | 0/10HPF | Consultation cases | Low-grade osteosarcoma？ | + | *COL1A1 exon1::USP6 exon2* | Local complete resection |
| 19 | MO | F | 60 | No | 1 | Painful mass | [Thigh](javascript:;) | 7.0 | Alive with tumor /25 | No | [Obvious](javascript:;) | ND | 3/10HPF | Consultation cases | MO | - | ND | Biopsy |
| 20 | MO | M | 21 | Yes (drainage) | 1 | Painful mass | [Inguinal](javascript:;) [region](javascript:;) | NA | NED/24 | No | [Obvious](javascript:;) | ND | 4/10HPF | Consultation cases | Extraskeletal osteosarcoma？ | - | ND | Local complete resection |
| 21 | MO | F | 80 | NA | 0.2 | Painless mass | Elbow | 4.0 | NA | No | [Obvious](javascript:;) | Desmin（-）、S100（-） | 2/10HPF | Consultation cases | Bone tumors？ | ND (tissue unavailable) | ND | Local complete resection |
| 22 | MO | M | 5 | No | 0.3 | Painful and swelling | Neck | 6.0 | NED/14 | Yes | [Obvious](javascript:;) | SMA（+）、SATB2（+）、MDM2（-）、Desmin（-） | 2/10HPF | Consultation cases | MO | + | *COL1A1 exon1::USP6 exon2* | Local complete resection |
| 23 | MO | F | 51 | NA | NA | NA | Chest wall | NA | NA | No | [Obvious](javascript:;) | SMA（+）、MSA（+）、Desmin（+）、Ki67 1～25%（+） | 1/10HPF | In-house case | Proliferative myositis  ？ fibromatosis？ | Failed | ND | Biopsy |
| 24 | MO | F | 38 | NA | 0.5 | Painless mass | [Thigh](javascript:;) | 17.0 | NA | Yes | Not [obvious](javascript:;) | ND | 3/10HPF | Consultation cases / In-house case | Osteosarcoma /MO | Failed | ND | Local complete resection |
| 25 | MO | M | 17 | NA | 2 | NA | [Thigh](javascript:;) | 6.0 | NA | No | Not [obvious](javascript:;) | ND | 3/10HPF | In-house case | Fibrous dysplasia？ | ND (decalcification) | ND | Biopsy |
| 26 | MO | M | 72 | NA | 60 | Painful and swelling | Shoulder | NA | NA | Yes | Not [obvious](javascript:;) | ND | 0/10HPF | In-house case | MO？ | Failed | ND | Local complete resection |
| 27 | MO | M | 37 | Yes (trauma) | 3 | Painful mass | Elbow | 8.0 | NA | No | Not [obvious](javascript:;) | ND | 1/10HPF | In-house case | Hemangioma with ossification？Osteoma? | ND (decalcification) | ND | Biopsy |
| 28 | MO | M | 25 | No | 2.7 | Painful mass | [Thigh](javascript:;) | 6.0 | NA | Yes | Not [obvious](javascript:;) | ND | 0/10HPF | In-house case | MO | ND (decalcification) | ND | Local complete resection |
| 29 | MO | M | 34 | No | 2 | Painless mass | Hip | 8.0 | NA | No | Not [obvious](javascript:;) | ND | 0/10HPF | In-house case | MO | ND (decalcification) | ND | Local complete resection |
| 30 | MO | M | 12 | No | 1 | Painful mass | Hip | NA | NED/78 | Yes | Not [obvious](javascript:;) | SMA（+）、MSA（+）、Desmin（-）、β-catenin（-）、Ki67 15％（+） | 20/10HPF | In-house case | Osteosarcoma？ | + | - | Local complete resection |
| 31 | MO | F | 15 | No | 2 | Painful mass | Arm | NA | NA | Yes | Not [obvious](javascript:;) | ND | 0/10HPF | In-house case | MO | ND  (off piece) | ND | Biopsy |
| 32 | MO | M | 22 | NA | 0.8 | Painful and swelling | [Thigh](javascript:;) | 10.0 | NED/68 | Yes | Not [obvious](javascript:;) | MDM2（-）、S-100（-）、Ki-67 10～15%（+） | 12/10HPF | In-house case | Extraskeletal osteosarcoma | + | *COL1A1 exon1::USP6 exon1* | Local complete resection |
| 33 | MO | M | 36 | No | 1 | Painful mass | [Thigh](javascript:;) | 7.0 | NED/67 | Yes | Not [obvious](javascript:;) | ND | 6/10HPF | In-house case | MO？ | + | ND  (poor RNA quality) | Local complete resection |
| 34 | MO | F | 13 | No | 8 | Painful mass | [Thigh](javascript:;) | 10.0 | [Recurrence](javascript:;)/40 | No | Not [obvious](javascript:;) | ND | 6/10HPF | In-house case | MO？ | ND (decalcification) | ND | Local complete resection |
| 35 | MO | F | 27 | No | 1 | Painful mass | [Thigh](javascript:;) | 3.0 | NA | No | Not [obvious](javascript:;) | ND | 3/10HPF | In-house case | [Giant-cell](javascript:;) [tumor](javascript:;)？ | ND (decalcification) | ND | Local complete resection |
| 36 | MO | M | 10 | Yes (swelling) | 0.7 | Painful mass | [Thigh](javascript:;) | 6.0 | NED/27 | No | [Obvious](javascript:;) | ND | 2/10HPF | In-house case | MO | ND (decalcification) | ND | Local complete resection |
| 37 | MO | M | 36 | NA | 1 | NA | [Thigh](javascript:;) | 3.7 | NA | Yes | Not [obvious](javascript:;) | SMA（+）、Desmin（-）、EMA（-）、  β-catenin（-）、MDM2（-）、S-100（-） | 0/10HPF | In-house case | MO | - | ND | Biopsy |
| 38 | MO | F | 31 | Yes (traction injury) | 3 | Painful mass | Hip | 2.7 | NED/24 | Yes | [Obvious](javascript:;) | ND | 0/10HPF | In-house case | Fibrous dysplasia？ | ND (decalcification) | ND | Local complete resection |
| 39 | MO | M | 7 | No | 2 | Painful mass | Hip | 3.0 | NED/21 | Yes | [Obvious](javascript:;) | ND | 3/10HPF | In-house case | MO | + | *UBE2G1 exon1::USP6 exon8-38* | Local complete resection |
| 40 | MO | M | 14 | Yes (sports injury) | 6 | Painful mass | [Thigh](javascript:;) | 5.2 | NED/9 | Yes | [Obvious](javascript:;) | ND | 1/10HPF | In-house case | MO | ND (decalcification) | ND | Local complete resection |
| 41 | MO | F | 54 | No | 0.8 | Painful mass | [Thigh](javascript:;) | 3.5 | NED/8 | No | Not [obvious](javascript:;) | ND | 5/10HPF | In-house case | MO？ | - | ND | Wide excision |
| 42 | MO | M | 15 | No | 2 | Painful mass | Hip | 5.0 | NED/90 | Yes | [Obvious](javascript:;) | SMA（+）、Desmin（-）、β-catenin（-）、S-100（-）、Ki67 12%（+） | 4/10HPF | In-house case | NF/FO？ | + | *MYH9 exon1::USP6 exon2* | Local complete resection |
| 43 | MO | M | 14 | No | 2 | Painful mass | crus | 4.0 | NED/1 | No | Not [obvious](javascript:;) | SMA（+）、SATB2（+）、S-100（-）、desmin（-）、Ki-67 20%（+）， | 1/10HPF | In-house case | NA | + | ND (poor RNA quality) | Biopsy |
| 44 | MO | F | 15 | No | 1 | Painful mass | [Thigh](javascript:;) | 4.0 | NED/1 | Yes | [Obvious](javascript:;) | SMA（+）、  S-100（-）、desmin（-）、Ki6710%（+） | 2/10HPF | In-house case | NA | + | ND(poor RNA quality) | Biopsy |
| 45 | FOPD | F | 35 | No | 36 | Painful mass | Toe | NA | NA | No | Not [obvious](javascript:;) | ND | 0/10HPF | Consultation cases | Fibroplasia？ | Failed | ND | NA |
| 46 | FOPD | M | 16 | NA | 0.3 | Painless mass | Finger | NA | NA | No | [Obvious](javascript:;) | ND | 2/10HPF | Consultation cases | NA | Failed | ND | NA |
| 47 | FOPD | F | 35 | NA | 2 | Painless mass | Finger | 0.5 | NA | No | Not [obvious](javascript:;) | ND | 0/10HPF | Consultation cases | Extraskeletal osteosarcoma | ND (tissue unavailable) | ND | Local complete resection |
| 48 | FOPD | M | 11 | NA | NA | NA | Finger | NA | NA | No | [Obvious](javascript:;) | ND | 0/10HPF | Consultation cases | FOPD | ND (tissue unavailable) | ND | Biopsy |
| 49 | FOPD | M | 64 | NA | 2 | Painless  mass | Finger | 0.8 | NA | No | Not [obvious](javascript:;) | ND | 5/10HPF | Consultation cases | FOPD | + | ND  (poor RNA quality) | Local complete resection |
| 50 | FOPD | F | 38 | Yes (trauma) | 2 | Painful mass | Finger | 1.9 | NED/42 | No | Not [obvious](javascript:;) | ND | 0/10HPF | Consultation cases | Chondrogenic tumor？ | ND (tissue unavailable) | ND | Local complete resection |
| 51 | FOPD | F | 44 | NA | NA | NA | Toe | NA | NA | Yes | Not [obvious](javascript:;) | ND | 3/10HPF | Consultation cases | Reactive lesion？ | ND (tissue unavailable) | ND | Biopsy |
| 52 | FOPD | M | 11 | Yes (bruise) | 1 | Painful and swelling | Finger | 1.5 | NED/124 | No | Not [obvious](javascript:;) | ND | 0/10HPF | In-house case | FOPD | Failed | ND | Local complete resection |
| 53 | FOPD | M | 31 | No | 2 | Painful mass | Finger | 3.0 | NA | No | Not [obvious](javascript:;) | ND | 0/10HPF | In-house case | [osteoma](javascript:;)？ | ND (decalcification) | ND | Local complete resection |
| 54 | FOPD | F | 61 | NA | 96 | Painless  mass | Finger | 1.5 | NA | No | [Obvious](javascript:;) | ND | 0/10HPF | In-house case | NA | ND (decalcification) | ND | Biopsy |
| 55 | FOPD | F | 12 | No | 12 | Painful mass | Toe | 3.0 | [Recurrence](javascript:;)/99 | No | Not [obvious](javascript:;) | ND | 0/10HPF | In-house case | O[steochondroma](javascript:;)？ | ND (decalcification) | ND | Partial mass resection |
| 56 | FOPD | M | 36 | Yes (trauma) | 12 | Painful mass | Finger | 1.5 | NA | No | Not [obvious](javascript:;) | ND | 0/10HPF | In-house case | MO | ND (decalcification) | ND | Local complete resection |
| 57 | FOPD | F | 27 | NA | 0.3 | Painful mass | Finger | 0.8 | NA | No | Not [obvious](javascript:;) | ND | 1/10HPF | In-house case | [Osteoblastoma](javascript:;)？ | Failed | ND | Biopsy |
| 58 | FOPD | M | 20 | Yes（skin damage） | 12 | skin damage | Finger | NA | NA | No | Not [obvious](javascript:;) | ND | 2/10HPF | In-house case | NA | + | *COL1A1 exon1::USP6 exon2* | Local complete resection |
| 59 | FOPD | M | 16 | No | 6 | Painless mass | Finger | 3.0 | [Recurrence](javascript:;)/16 | No | [Obvious](javascript:;) | ND | 0/10HPF | In-house case | FOPD | ND (decalcification) | ND | Partial mass resection |
| 60 | ST-ABC | M | 14 | Yes  (sport injury) | 3 | Painful mass | Hip | 5.0 | NED/9 | Yes | Not  [Obvious](javascript:;) | SMA（+）、SATB2（+）、Desmin（-）、S100（-）、MDM2（-） | 1/10HPF | In-house case | Fibroskeletal lesions? | + | *COL1A1 exon1::USP6 exon1* | Local complete resection |
| 61 | ST-ABC | M | 15 | No | 2 | Painful mass | Lumbar vertebral side | 4.0 | NED/3 | No | Not [Obvious](javascript:;) | SMA（+）、SATB2（+）、S-100（-）、Desmin（-）、MDM2（-） | 0/10HPF | In-house case | NA | + | *COL1A1 exon1::USP6 exon2* | Local complete resection |
| 62 | FO | F | 23 | No | 1 | Painful mass | Neck | 2.0 | Alive with tumor /70 | No | [Obvious](javascript:;) | SMA（+）、MSA（+）、S-100（-）、Desmin（-）、Ki-67 15～20%（+） | 4/10HPF | Consultation cases | Fibromatosis | Failed | ND | Biopsy |
| 63 | FO | F | 44 | No | 2 | Painful mass | Thigh | 12.0 | [Recurrence](javascript:;)/26 | Yes | [Obvious](javascript:;) | SMA（+）、MSA（+）、S-100（-）、Desmin（-）、MDM2（-）、Ki-67 5-20%（+） | 10/10HPF | Consultation cases | FO? Osteosarcoma? | + | *COL1A1 exon1::USP6 exon3* | Local complete resection |
| 64 | FO | M | 28 | No | 0.5 | Painful mass | [Infraclavicularis](javascript:;) | 4.0 | NED/15 | Yes | [Obvious](javascript:;) | SMA（+）、MSA（+）、Desmin（-）、SATB2（+）、β-catenin（-）、S-100（-）、MDM2（-） | 5/10HPF | Consultation cases | MO？ | + | *COL1A1 exon1::USP6 exon2* | Wide excision |
| 65 | FO | M | 10 | No | 0.2 | Painful mass | Knee | 5.0 | NED/9 | No | [Obvious](javascript:;) | SMA（+）、SATB2（+）、Desmin（-）、Ki-67 15%（+） |  | Consultation cases | Extraskeletal osteosarcoma? | + | *COL1A1 exon1::USP6 exon2* | Local complete resection |
| 66 | FO | M | 32 | No | 1 | Painful mass | Arm | 2.0 | NED/2 | No | [Obvious](javascript:;) | SMA（+）、SATB2（+）、MSA（-）、Desmin（-）、S-100（-）、  β-catenin（-）、Ki-67 3-5%（+） | 5/10HPF | Consultation cases | NA | + | *SNHG3 exon1::USP6 exon8-38* | NA |
| 67 | FO | F | 2 | NA | 0.5 | Swelling | Thigh | 6.5 | NED/127 | No | [Obvious](javascript:;) | ND | 0/10HPF | In-house case | MO | Failed | ND | Local complete resection |
| 68 | FO | M | 42 | NA | 5 | Painful and swelling | Leg | 20.0 | NA | No | [Obvious](javascript:;) | ND | 2/10HPF | In-house case | [Benign](javascript:;) [lesion](javascript:;) | Failed | ND | Local complete resection |
| 69 | FO | F | 26 | No | 1 | Painful mass | Thigh | 5.0 | NED/47 | Yes | Not [obvious](javascript:;) | ND | 6/10HPF | In-house case | Fibrous dysplasia? | ND (decalcification) | ND | Local complete resection |
| 70 | FO | M | 24 | No | 2 | Painful and swelling | Thigh | 15.0 | NA | No | Not [obvious](javascript:;) | ND | 0/10HPF | In-house case | MO？ | ND  (off piece) | ND | Biopsy |
| 71 | FO | M | 18 | No | 0.2 | Painful mass | Knee | 5.0 | NED/26 | Yes | [Obvious](javascript:;) | SMA（+）、SATB2（+）、S-100（-）、Desmin（-）、MDM2（-）、Ki-67 1~10%（+） | 2/10HPF | In-house case | Reactive lesion? | + | *COL1A1 exon1::USP6 exon2* | Local complete resection |
| 72 | FO | F | 36 | No | 3 | Painful mass | Thigh | 5.0 | NED/12 | No | [Obvious](javascript:;) | ND | 2/10HPF | In-house case | [Benign](javascript:;) [lesion](javascript:;) | + | *COL1A1 exon1::USP6 exon2* | Wide excision |
| 73 | FO | F | 35 | No | 72 | Painful mass | Thigh | 7.0 | NA | No | [Obvious](javascript:;) | ND | 1/10HPF | In-house case | Fibrohistiocytoma？ | Failed | ND | Local complete resection |

MO, myositis ossificans; FOPD, fibro-osseous pseudotumor of digits; ST-ABC, soft tissue aneurysmal bone cyst; FO, fasciitis ossificans; NF, nodular fasciitis; F, female; M, male; mo, month; FISH, Fluorescence in situ hybridization; RT-PCR, Reverse Transcription-Polymerase Chain Reaction; NGS, Next-generation sequencing; NA, not available; ND, not done; NED, no evidence of disease
